# Detection and Analysis of Human Papillomavirus (HPV) DNA in Breast Cancer Patients by an Effective Method of HPV Capture

**DOI:** 10.1371/journal.pone.0090343

**Published:** 2014-03-10

**Authors:** Ting Wang, Xi Zeng, Weiyang Li, Haijun Zhu, Guan Wang, Xiao Liu, Yonggang Lv, Jinghua Wu, Xuehan Zhuang, Juliang Zhang, Yi Zhao, Haodong Huang, Jing Fan, Qing Yao, Chenyang He, Xiuqing Zhang, Chen Huang, Jianghao Chen, Ling Wang

**Affiliations:** 1 Department of Vascular and Endocrine Surgery, Xijing Hospital, Fourth Military Medical University, Xi An, China; 2 BGI-Shenzhen, Shenzhen, China; 3 Department of Nephrology, Xijing Hospital, Fourth Military Medical University, Xi An, China; 4 School of Bioscience and Bioengineering, South China University of Technology, Guang Zhou, Chinah; 5 Division of Genomics and Bioinformatics, CUHK-BGI Innovation Institute of Trans-omics, The Chinese University of Hong Kong, Hong Kong, China; 6 Department of Biology, University of Copenhagen, Copenhagen, Denmark; International Centre for Genetic Engineering and Biotechnology, Italy

## Abstract

Despite an increase in the number of molecular epidemiological studies conducted in recent years to evaluate the association between human papillomavirus (HPV) and the risk of breast carcinoma, these studies remain inconclusive. Here we aim to detect HPV DNA in various tissues from patients with breast carcinoma using the method of HPV capture combined with massive paralleled sequencing (MPS). To validate the confidence of our methods, 15 cervical cancer samples were tested by PCR and the new method. Results showed that there was 100% consistence between the two methods.DNA from peripheral blood, tumor tissue, adjacent lymph nodes and adjacent normal tissue were collected from seven malignant breast cancer patients, and HPV type 16(HPV16) was detected in 1/7, 1/7, 1/7and 1/7 of patients respectively. Peripheral blood, tumor tissue and adjacent normal tissue were also collected from two patients with benign breast tumor, and 1/2, 2/2 and 2/2 was detected to have HPV16 DNA respectively. MPS metrics including mapping ratio, coverage, depth and SNVs were provided to characterize HPV in samples. The average coverage was 69% and 61.2% for malignant and benign samples respectively. 126 SNVs were identified in all 9 samples. The maximum number of SNVs was located in the gene of E2 and E4 among all samples. Our study not only provided an efficient method to capture HPV DNA, but detected the SNVS, coverage, SNV type and depth. The finding has provided further clue of association between HPV16 and breast cancer.

## Introduction

Breast cancer is the second most common type of cancer worldwide, with the highest prevalence rate among women in the world and 1/8 of women suffer from breast cancer during lifetime [1]. Hereditary mutations (such as BRAC1/2, TP53, PTEN, and STK11) cause only about 5% of breast cancer case [2]. Infectious factors was responsible for 18% of human cancers and it is well accepted that human breast cancer is highly associated with environmental factors, such as diet, source of water, virus, radiation [3], [4], [5], [6]. Breast tumorigenesis might be promoted by viral infection. High-risk HPV such as HPV type 16(HPV16), HPV type 18(HPV18) were associated with cervical cancer, anogenital cancers and cancers of other organs [7], [8]. Factors that favor a small proportion of HPV16 infections to progress to cancer are still poorly understood, but the genetic variation has implicated a role of HPV16 in previous study [9]–[10].

There were increasing studies reporting on the involvement of HPV DNA in breast cancer in recent years, but the conclusions remained to be highly controversial. Di Lonardo et al. [11] first reported the detection of HPV16 DNA in 29% of 17 patients with breast cancer by polymerase chain reaction (PCR). De Villiers et al. [12] found HPV DNA in 86% of breast cancers (25/29) and in 69% of the corresponding nipple sample (20/29). HPV11 was detected as the most prevalent type, followed by HPV type 6. Only 12% of the samples displayed signal of HPV16, while HPV18 was failed to be found in all samples. HPV infection was also reported in breast cancer by some other studies [13]–[15]. However, several other studies failed to detect any signals of HPV in breast cancer cells [16]–[17]. Beyond that, Wrede et al. [18] recruited 95 women suffering from breast cancer and screened HPV 6b, 11, 13, 16, 18, 30, 31, 32, 33, 45, and 51. , but they didn't find any signal of HPV infection.

Previous studies were mostly based on PCR based approach, in which unique primers targeting HPV genes were designed to screen the existence of virus DNA. This strategy is easy to access and screen in large number of samples, but limited in sensitivity and specificity, thus probably contribute to so many controversial studies. Here, we adopted the sequence capture and MPS method to detect the signal of HPV DNA fragment which also provided unique opportunity to study HPV variations in its whole genome level. The study validated the efficiency of the novel method in breast cancer and discovered HPV DNA can be detected in blood, tumor tissue, adjacent lymph nodes and adjacent normal tissue of breast cancer patient, furthermore analyze the variations among them.

In this study, we not only provided an efficient method to capture HPV DNA in tissues of breast cancer patients, but also found HPV SNVs difference in the samples. This provided new insight in studying the association between HPV infection and breast cancer.

## Material and Methods

### Material

9 sets of breast cancer samples were collected respectively from 9 female patients and received surgery at the department of vascular and endocrine surgery, Xijing hospital, the Fourth Military Medical University, China, between October 2010 and October 2011 (Table S1). Exclusion criteria were: 1. systemic therapy prior to surgery, 2. bilateral breast cancer, 3. metastatic or recurrent disease, 4. cancer of other origin. Adjuvant therapy was based on current NCCN (National Comprehensive Cancer Network) guidelines. In addition, In order to evaluate the accuracy of our method, 15cervical cancer samples were also collected from Xijing hospital (Table S2). Written Informed consents were obtained from each patient. All aspects of this study were reviewed and approved by the institutional review board at the Xijing Hospital and BGI.

The 9 sets of samples were respectively from 7 malignant breast cancer patients whose ages ranged from 37 to 85 and 2 benign breast cancer patients. For malignant breast cancer, each set included 4 types of tissues (tumor tissue, adjacent normal tissue, lymph nodes and blood). For benign patients, each set included 3 types of tissues (tumor tissue, para-carcinoma tissue and blood). The blood samples were collected before surgery and the tissue samples were preserved in liquid nitrogen with programmed freezing in 1 hour after isolation. All samples were used to detect signals of HPV DNA.

All 34 read datasets are accessible through the NCBI Short Read Archive (http://www.ncbi.nlm.nih.gov/Traces/sra/) under the accession number SRA124368 (SRP035570).

### HPV probes design and HPV genome enrichment and sequencing

Full-length HPV genome of 17 types (6,11,16,18,31,33,35,39,45,52,56,58,59,66,68,69,82)were used to design the HPV probes by MyGenostics (MyGenostics, Baltimore, MD). Sequencing libraries of 170 bp insert size were constructed following the instruction of Illumina. Genomic DNA was sheared to around 150 bp–200 bp DNA fragments by Covaris E-210 (Covaris, Inc., Woburn, MA). These fragments were purified, end blunted, “A” tailed, and adaptor ligated. 10 cycles of PCR were performed after size selection in 2% agarose gel. The concentration of libraries was quantified by Bioanalyzer 2100 (Agilent Technologies, Santa Clara, CA).The hybridization process was carried according to MyGenostics GenCapTM Target Enrichment Protocol. Libraries were hybridized with HPV probes(Including 17 high-risk types of HPV) at 65°C for 24 hours and then washed to remove un-captured fragments. The eluted fragments were amplified by 18 cycles of PCR to generate libraries for sequencing. Libraries were quantified and preceded to 101 cycles' paired-end index sequencing using the Illumina HiSeq 2000 sequencer according to manufacturer's instructions (Illumina Inc., San Diego, CA).

### HPV Fragment detection

**Figure 1:**
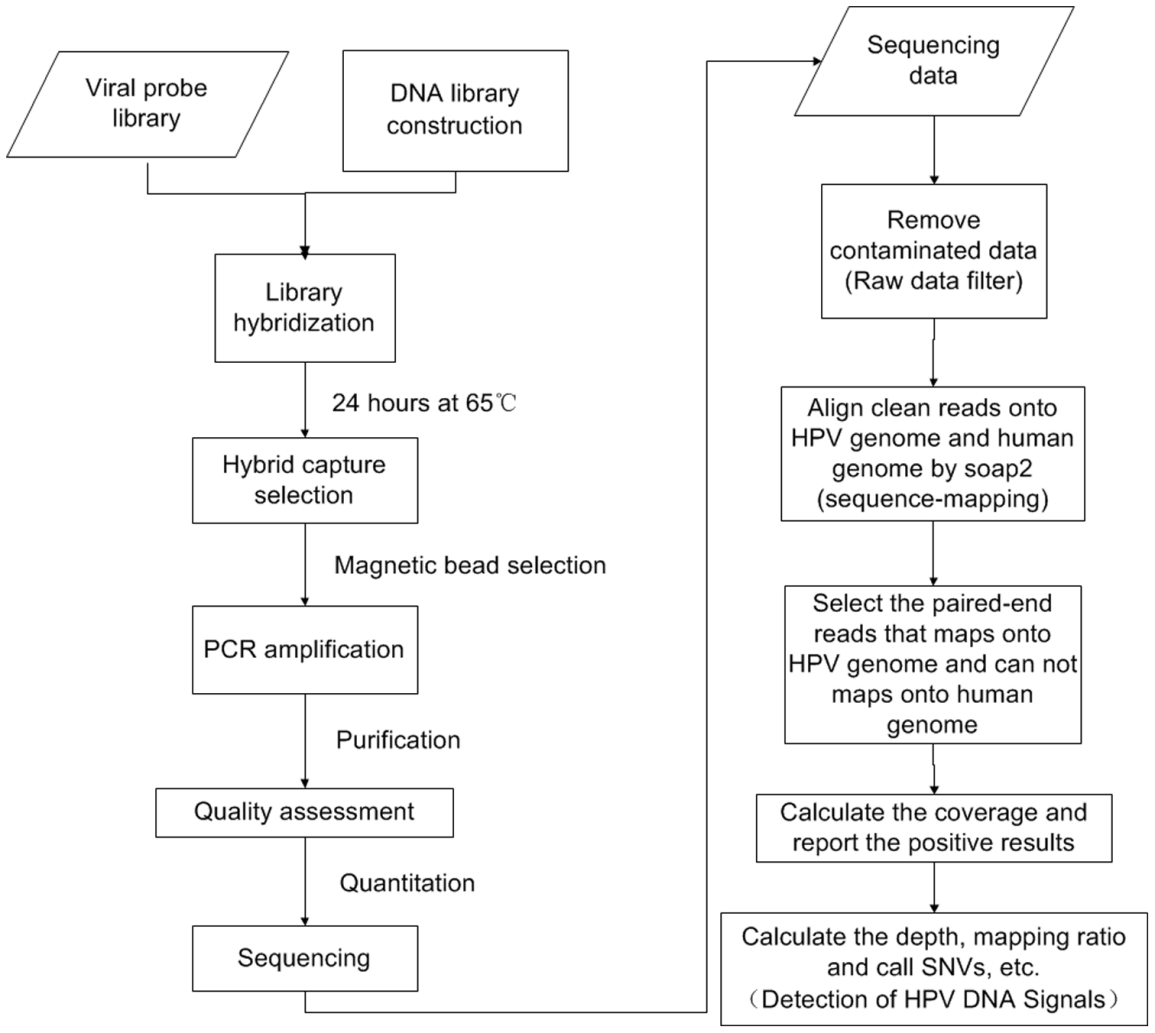
Overview of the workflow. It shows the pipeline of experimental process and bioinformatics process for this study.

The 100 bp paired-end reads were preceded into bioinformatics process. The paired-end reads which contained the signals of HPV were picked out, and then the mapping ratio, depth, coverage and SNVs on HPV genome of these reads would be obtained. The details of the bioinformatics process are presented as follows (Figure 1).

#### Raw data filter

Low quality reads (a read with more than 50% low quality value bases whose quality value is less than 5) and duplication reads, as well as adaptor contaminated reads, were firstly removed. The remained clean reads were obtained for subsequent analysis.

#### Sequence Mapping

Clean reads were mapped to human genome (NCBI build 37, HG19) and HPV genome (6,11,16,18,31,33,35,39,45,52,56,58,59,66,68,69,82) using SOAP2 (-l 40 -v 5 -r 1) [19].

#### Detection of HPV DNA Signals

If a paired-end reads were not able to map onto human genome but were able to map onto HPV genome, it would be reported as signals of HPV DNA for subsequent analysis.

The coverage, depth and mapping ratio on HPV genome were calculated based on these reported reads. A sample was considered HPV positive if the Coverage of the sample on HPV genome was higher than 50%.
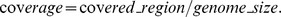



Covered_region means the size of the covered region on HPV genome by reported reads; genome_size here means the size of the HPV genome. We also calculated average depth and normalized depth.
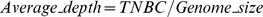


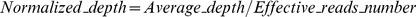



*TNBC* means the number of bases mapped on HPV genome. Effective reads number means the total number of reads after the process of *Raw data filter*.

For one certain sample, the depth reflects the reads number covered on the HPV16 genome in raw data. Normalized depth was defined to make the depth comparable among samples with different data quantities.

The SNV of HPV genome was called based on the alignment results of the reported reads using soapsnp (-r 0.0005 -e 0.001 -t -u -L 100) [20].

The criterions of quality control of SNVs calling were: *1.The Quality score of consensus genotype* is not smaller than 20; 2. *Count of uniquely mapped second best base*/*Count of uniquely mapped best base* >0.2; 3. *Count of uniquely mapped second best base is not* less than 2.

## Results

### HPV detection in cervical cancer samples

It's well accepted that HPV plays an important role in cervical cancer. We obtained 15 cervical cancer samples which included 5 negative samples and 10 positive samples by PCR diagnosis. We carried on the HPV capture in these 15 samples, and found 100% accuracy in the result with our method. (Table S2)

### Overview of HPV detection in breast cancer

We analyzed 9 sets of samples collected respectively from 7 malignant breast cancer patients and 2 benign breast tumor patients. HPV16 (K02718.1) was the only HPV type detected in all of the samples. For malignant breast cancer, HPV16 was found only in the set of samples from patient C080. We also found HPV16 DNA in 1/2, 2/2, 2/2 of patients with benign breast tumor, in blood, tumor and adjacent normal tissue respectively. MPS metrics and clinical data were reported in Table S1.

Additionally, we tried to detect the HPV integrations using the method of HIVID [21], but all samples showed negative results.

### SNVs of HPV16 identification in various samples

To further analyze any discrepancies among various tissues, we tried to identify the SNV pattern of HPV16 in all the samples (Table S3). For the set samples of C080, the normal and blood tissue had more SNVS in the region of E2, E4, L1 and L2. For the set samples of T009 and T007, the normal and tumor tissue had more SNVS in these regions. The region of E2 and E4 had more SNVS among all samples (Table S4).

Then we surveyed the SNV number of transition and transversion in different tissue types of all patients. Four tissue types of C080 had the same trend that there were more transversions than transitions. The normal and tumor tissue of T009 had the contrary trend with set samples of C080(Table 1). Then we surveyed the synonymous and non-synonymous SNVs (Table S5). Most of the SNVs belonged to the non-synonymous mutations. The proportion of nonsynonymous mutations in genes of L1,L2,E1,E2,E5 and E6 was 76%,75%,93%,75.7%,100%,100%, While the proportion of synonymous mutation in genes of E7 and E4 was 80%,100%.The SNVS number of transition and transversion was 30 and 57 among the SNVS of nonsynonymous (Table 2),but was 47 and 6 in synonymous mutations. It had the similar trend that the number of synonymous mutations was less than the non-synonymous mutations in benign and malignant samples (Table S6).

**Table 1:** SNV Information.

Sample ID	Transition	Transversion	Total
C080	11	13	24
C080C	4	5	9
C080L	3	6	9
C080N	7	13	20
T007C	9	6	15
T007N	6	9	15
T009	1	8	9
T009C	7	6	13
T009N	7	5	12
Total	55	71	126

This table revealed that the distribution of SNVS of transition and transversion in different samples.

**Table 2:** Summary of the polymorphisms in HPV genes.

Gene	Polymorphic sites	Nonsyn^1^			Syn^2^			Gene size(bp)
		Ts^3^	Tv^4^	Nonsyn(%)	Ts	Tv	Syn(%)	
E6	10	1	9	100	0	0	0	477
E7	10	1	1	20	7	1	80	297
E1	15	4	10	93.33	0	1	6.66	1949
E2	37	22	6	75.67	9	0	24.32	1098
E4	26	0	0	0	26	0	100	288
E5	5	0	5	100	0	0	0	252
L2	20	0	15	75	3	2	25	1422
L1	17	2	11	76.47	2	2	23.52	1596

1 nonsynonymous mutation.

2 synonymous mutation.

3 Transition.

4 Transversion.

**Figure 2:**
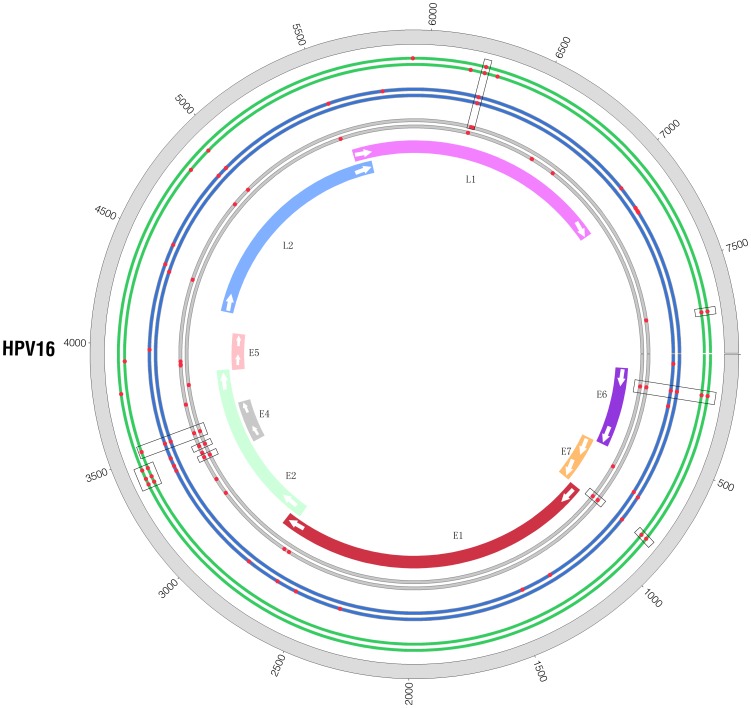
Contrast of SNVs between benign samples and malignant samples. There are three pairs of circles close together in this figure. The color of green, blue and grey represents the tumor tissue, blood and adjacent normal tissue respectively. In every pair of circle, the inner circle represents malignant sample C080 and the outer circle represents benign sample T009. Each red point is the position of a SNV and the rectangular black box surround the SNVs which share the same position in several samples.

We also performed comparisons between malignant and benign samples for the same tissue type and found that almost every tissue type from malignant sample set C080 and benign sample set T009 shared some common SNVs (Figure 2, Table S7).

**Figure 3:**
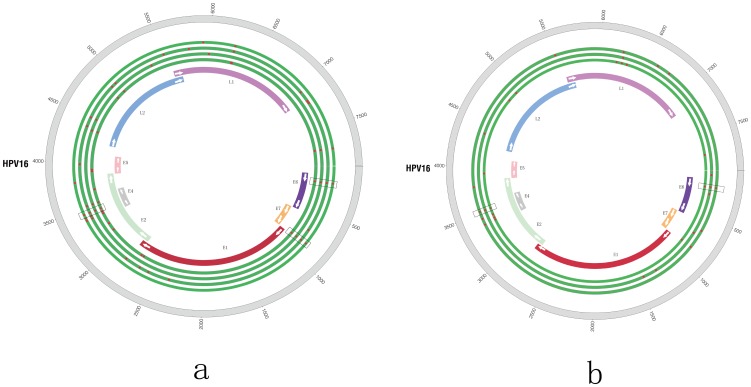
The display of SNVs in a sample set. **A**. SNVs for sample set of C080; **B**. SNVs for sample set of T009. The locations of the genes are shown in different colors. Each red point is the position of a SNV and the rectangular black box surround the SNVs which share the same position in several samples. Genomic positions are numbered. **A**. It is cancer tumor, blood, lymph nodes and adjacent normal tissue from the outside to the inside. **B**. There are three green circles and it is tumor tissue, blood and adjacent normal tissue from the outside to the inside.

In patient C080 with malignant tumor, the numbers of SNV were 24, 9, 20 and 9, respectively in blood, tumor tissue, and adjacent normal tissue and lymph tissues. 3 common SNVs were identified in all tissues which were located in the position 178(E6, Nonsynonymous), 846(E7, Synonymous), 3448(E2, Nonsynonymous; E4, Synonymous) of HPV genome (Figure 3a). Accordingly, in patient T009 with benign tumor, the numbers of SNV were 9, 13 and 12 respectively in blood, tumor and adjacent normal tissue. All tissues shared 2 SNVs located in the position 178(E6, Nonsynonymous), 3523(E4, E2, Synonymous) of HPV genome (Figure 3b). Besides, the numbers of SNV were both 15 in two tissue types (tumor, adjacent normal) of T007, while most of common SNVs were located in the region of 2000 bp–4500 bp.

To pinpoint the SNV variations among the different tissue types in C080, we compare the tumor SNVs with other tissues and identify one SNV was tumor specific (5926, L1, Synonymous).

## Discussion

There are enough evidences for the relationships between HPV and cervical cancer [22], but the relationship between HPV and breast cancer still remains elusive. Even in the study with positive results, the detected subtypes tend to be various. Many studies suggest viral oncogenesis as an etiological factor for breast cancer, while there are also studies coming out of negative result for HPV. Thus it remains controversial.

The PCR based approach has dominated the previous molecular investigations of HPV in breast cancer, and made significant contributions in some discoveries. Although convenient in common molecular laboratories, it lacks enough sensitivity for further sequence analysis and stability which to some extent revealed by so many controversial reports.

MPS technology has been widely used to study the origin, selection and evolution of virus in recent years. The novel method we introduced here combined MPS with virus-target probe capture, as well as high-performance bioinformatics algorithm which are capable to detect signals of target virus and analyze its sequences. MPS makes large-scale paralleled detection of virus signal possible and enables us to reconstruct the virus genome in base-pair resolution, and the capture technology greatly enriched the interested region thus reduced the cost. Our method is not only able to detect virus signals in multiple tissues, but also has the ability to detect the signals of multiple viruses at the same time in a single run with high specificity and sensitivity. To avoid the potential false positive signals introduced by contamination reads, we set strict filtering of 50% of HPV genome is covered by at least one read for a sample to be called as HPV positive. This cut off was adopted to judge the existence of HPV in our 15 cervical tumors, and showed a 100% consistency with PCR result.

HPV16 DNA was detected in several tissue types of both malignant and benign samples from breast cancer patients using our new method. Analyzing the coverage, depth distribution, mapping ratio and SNVs of virus genome are helpful for us to find some clue about the association between HPV16 and breast cancer.

Traditionally HPV was thought to only infect cutaneous and mucosal tissues but not transmissible via blood. Some discoveries of HPV DNA in blood had brought this notion into question. Chen AC. et al. [23] screened HPV DNA in peripheral blood by PCR in 180 healthy male blood donors in age from 18–76 and found 15 donors (5.8%) with positive result. In another study Andreas Widschwendter et al. also found HPV16 DNA in blood (5/11) from breast cancer patient [24]. In our study, HPV16 DNA was detected in blood of both malignant breast cancer patient (1/7) and patient with benign tumor (1/2).Although we detected HPV DNA in blood of breast cancer patient, the role of HPV16 remains to be elucidated.

The tissue type which included more SNVS had significant difference between malignant and benign samples. More SNVS were included in the blood and normal tissue of C080, while the tumor and normal tissues of T007 and T009 included more SNVs. Most of SNVS were located in the E2 region. The papillomavirus E2 protein is required for viral replication and regulates both viral transcription and replication, and therefore plays a central role in the viral life cycle. In addition, E2 is also important for repressing oncoprotein transcription. The mutation on E2 region may have an effect on these aspects in order to keep adaptive [25]–[26].

Furthermore, common SNVs of malignant cancer sample set C080 (E6, 178, nonsynonymous; E7, 846, synonymous; E2, 3448, nonsynonymous) and that of benign tumor sample set T009 (E6, 178, nonsynonymous; E2, 3523, synonymous) were significantly discrepant. The SNVS of 178,846 had been identified as the most frequent sequence variation site by previous studies and they had been identified as markers of the Asian lineage [27], [28].Previous study has also found the discrete regions of 647–846 show the most frequently observed substitution in HPV16 E7 open reading frame [29]. When compared HPV SNV patterns of different tissues from the same patient (Figure 3a, Figure 3b), we identified the SNV (L1, 5926,synonymous) in C080C.The SNV of 5926 which only existed in the C080C could not lead to change of the protein coding.

We carried on the SNVS categorization according to the synonymous and non-synonymous mutations. The total SNVS number was 126. We got more than 126 SNVS when performing the analysis of synonymous and nonsynonymous mutations. Some SNVS located in two genes region was the cause of the phenomenon. For example the SNVS located in the E4 gene could also located in the E2 gene. The synonymous mutations do not change the protein coding and are not affected by natural selection,while nonsynonymous mutations alter the protein sequences and can be affected by natural selection [30]–[31]. Most of the nonsynonymous mutations were located in genes of L1, L2, E1 and E2.The genes of L1 and L2 encode the structural capsid proteins, and mutations of these genes may indicate that these amino acid changes are beneficial to accommodate the human papillomavirus to its environment [32]. For example SNV of position 6241(L1, Nonsynonymous) existed in six samples (C080, C080C, C080N, T007N, T009, T009C), which changed the protein coding. This change might lead to selective advantage of HPV16 for escaping from immune recognition by the host immune system. The ability of E1 and E2 to complex with each other appears to be essential for efficient viral DNA replication and E6 gene of the high risk group is known to be oncogene [25], [26].These mutations of the three genes may have the association with keeping functional advantage. Viral gene E4 expression reflects viral replication, and E7 is known as oncogene [28].The synonymous mutations were inclined to the two genes. The changes may keep the adaption of HPV16 by base substitution. The SNV pattern was more inclined to the transition in the synonymous mutations, but the pattern was more inclined to the transversion in the nonsynonymous mutations. The phenomenon had the similar trend with previous study [33].

Improvements of awareness in patient and physician about necessity in treatment for HPV infection might be the preferred strategy for clinical prevention. Wang T et al. [34] proposed that prophylactic HPV vaccines for cervical cancer may also reduce the development of breast cancer in women and the repression of viral oncogene expression can prevent the growth or survival of breast cancer cells.

In our study, we not only found several samples which had high coverage and depth of HPV, but also detected significant SNVs difference in distribution, number and type. The finding implies that a possible causal role of HPV infection in breast cancer carcinogenesis could not be ruled out. We believe the finding has provided further clue of association between HPV16 and breast cancer. Certainly, there is a need of further study to confirm the role of HPV16 in breast cancer.

## Supporting Information

Table S1 Summary of clinical data and sequencing analysis.
(XLSX)

Table S2 Supplementary Results and validation of 15 cervical cancer samples.
(XLS)

Table S3 SNVs of all the HPV positive samples.
(XLS)

Table S4 The distribution of SNV in HPV gene.
(XLSX)

Table S5 The information of whether the SNV is synonymous or nonsynonymous.
(XLS)

Table S6 The distribution of SNVS of Nonsynonymous and synonymous in benign and maligant samples.
(XLS)

Table S7 The common SNVs of the same tissue type.
(XLS)
